# Esmethadone (REL-1017) and Other Uncompetitive NMDAR Channel Blockers May Improve Mood Disorders via Modulation of Synaptic Kinase-Mediated Signaling

**DOI:** 10.3390/ijms232012196

**Published:** 2022-10-13

**Authors:** Stephen M. Stahl, Sara De Martin, Andrea Mattarei, Ezio Bettini, Luca Pani, Clotilde Guidetti, Franco Folli, Marc de Somer, Sergio Traversa, Charles E. Inturrisi, Marco Pappagallo, Marco Gentilucci, Andrea Alimonti, Maurizio Fava, Paolo L. Manfredi

**Affiliations:** 1 Department of Psychiatry, VAMC (SD), University of California, La Jolla, San Diego, CA 92093, USA; 2 Neuroscience Education Institute, Carlsbad, CA 92008, USA; 3 Department of Pharmaceutical and Pharmacological Sciences, University of Padua, 35122 Padua, Italy; 4 In Vitro Pharmacology Department, Aptuit, An Evotec Company, 37135 Verona, Italy; 5 Department of Psychiatry and Behavioral Sciences, School of Medicine, University of Miami, Miami, FL 33146, USA; 6 Department of Biomedical, Metabolic and Neural Sciences, University of Modena and Reggio Emilia, 41121 Modena, Italy; 7 Relmada Therapeutics, Coral Gables, FL 33134, USA; 8 Child and Adolescent Neuropsychiatry Unit, Department of Neuroscience, Bambino Gesù Pediatric Hospital, 00165 Rome, Italy; 9 Department of Health Sciences, University of Milan, 20122 Milan, Italy; 10 MGGM LLC, 85 Baker Road, Kerhonkson, NY 12446, USA; 11 Institute of Oncology Research, Southern Switzerland, 6500 Bellinzona, Switzerland; 12 The Institute of Oncology Research, Università della Svizzera Italiana, 6500 Bellinzona, Switzerland; 13 Veneto Institute of Molecular Medicine, 35129 Padua, Italy; 14 Department of Medicine, Zurich University, 8006 Zurich, Switzerland; 15 Department of Medicine—DIMED, University of Padua, 35122 Padua, Italy; 16 Department of Psychiatry, Massachusetts General Hospital, Boston, MA 02114, USA

**Keywords:** depression, dextromethorphan, d-methadone, esmethadone, esketamine, ketamine, major depressive disorder, REL-1017, neural plasticity, N-methyl-D-aspartate receptor

## Abstract

This article presents a mechanism of action hypothesis to explain the rapid antidepressant effects of esmethadone (REL-1017) and other uncompetitive N-methyl-D-aspartate receptor (NMDAR) antagonists and presents a corresponding mechanism of disease hypothesis for major depressive disorder (MDD). Esmethadone and other uncompetitive NMDAR antagonists may restore physiological neural plasticity in animal models of depressive-like behavior and in patients with MDD via preferential tonic block of pathologically hyperactive GluN2D subtypes. Tonic Ca^2+^ currents via GluN2D subtypes regulate the homeostatic availability of synaptic proteins. MDD and depressive behaviors may be determined by reduced homeostatic availability of synaptic proteins, due to upregulated tonic Ca^2+^ currents through GluN2D subtypes. The preferential activity of low-potency NMDAR antagonists for GluN2D subtypes may explain their rapid antidepressant effects in the absence of dissociative side effects.

## 1. Introduction: Impaired Neural Plasticity and Major Depressive Disorder

Neural plasticity impairment caused by altered glutamatergic signaling has recently emerged as a mechanism of disease hypothesis for mood disorders, superseding the classic serotonergic hypothesis [1,2,3,4]. Treatment with standard antidepressants results in partial and delayed responses in a subset of patients [5], and their benefits are increasingly questioned [6]. Patients with major depressive disorder (MDD) suffer not only from depressed mood but also from cognitive deficits that can be related to the impairment of neural plasticity [7]. Chronic inescapable stress and other models of depressive-like behavior affect hippocampal neural plasticity [8]. Patients with MDD have decreased hippocampal volume [9]. These neuropathological and neuroimaging findings may be correlated to a decrease in neuronal arborization, including reduced synaptic spine volume and impaired spinogenesis, seen in animal models of depressive-like behavior [10,11,12].

## 2. N-Methyl-D-Aspartate Receptors Regulate Neural Plasticity

Neural plasticity is regulated by glutamatergic signaling via NMDARs [13,14]. NMDARs are heterotetramers composed of two constitutive glycine-binding NR1 subunits necessary for membrane expression of the functional receptor and two regulatory glutamate-binding NR2 subunits named 2A, 2B, 2C, and 2D, which differentially regulate Ca^2+^ influx across the NMDAR channel. NMDAR subtypes have distinct regional and developmental distributions [14]. NMDAR subtypes GluN2A, GluN2B, GluN2C, and GluN2D each differentially regulate Ca^2+^ influx across the neuronal membrane during both phasic and tonic receptor activity. NMDAR subtypes containing the 3A and 3B subunits bind only glycine, not glutamate, and are not blocked by Mg^2+^ [15,16]. While their synaptic expression may also serve to modulate neuronal Ca^2+^ signaling, the NMDAR subtypes containing 3A or 3B subunits will not be discussed in this review. G protein–coupled glutamate receptor signaling also plays a role in neural plasticity; however, this review is limited to an attempt to understand the direct downstream consequences of the regulation of ionotropic glutamatergic Ca^2+^ signaling via uncompetitive NMDAR antagonists. Therefore, G protein–coupled glutamate receptors will not be discussed.

## 3. Clinical Uses of Uncompetitive NMDAR Antagonists

Uncompetitive NMDAR antagonists are a relatively recently described class of molecules with expanding clinical applications. The uncompetitive NMDAR antagonists memantine, dextromethorphan (combined with quinidine), and esketamine (the (+)-enantiomer of ketamine) have been approved by the U.S. Food and Drug Administration (FDA) to treat Alzheimer’s disease, pseudobulbar affect, and treatment-resistant depression (TRD), respectively. The dextromethorphan-bupropion combination as well as esmethadone have shown rapid, robust, and sustained efficacy for MDD in Phase 2 clinical trials and have advanced to Phase 3 development [17,18,19]. The dextromethorphan-bupropion combination was recently FDA-approved for the treatment of MDD. Recent studies have improved our understanding of the interactions between NMDAR antagonists and their binding sites in the receptor channel and the potential clinical implications of the pharmacological differences among these molecules [20].

Ketamine is an uncompetitive NMDAR antagonist in use as an anesthetic administered intravenously that was serendipitously found to have rapid antidepressant effects, later confirmed in controlled clinical trials [21]. The antidepressant effects of intravenous (IV) ketamine were replicated with intranasal esketamine [22,23], leading to FDA approval of intranasal esketamine as a rapidly acting antidepressant for TRD. The effects of uncompetitive NMDAR antagonists in reversing depressive-like behavior and restoring neural plasticity in animal models were first observed with MK-801 (dizocilpine) [24]. In the case of MK-801, uncompetitive NMDAR blockade resulted in a profound alteration of physiological NMDAR activity due to the high potency of MK-801, causing severe adverse effects and precluding clinical applications. Ketamine is less potent compared to MK-801; however, the rapid, robust, and sustained antidepressant effects of ketamine and esketamine, at the doses currently in use to treat patients with MDD, are accompanied by short-lived dissociative effects in over 70% of patients, requiring clinical observation after administration [25].

The initial hypotheses for the antidepressant effects of ketamine were made under the assumption that temporary dissociative effects were integral to the therapeutic effects. However, the induction of dissociative effects by ketamine and esketamine may not be necessary for their rapid antidepressant effects [26], and, in fact, low doses of IV ketamine (0.1 mg/kg) were found to be therapeutic without dissociative effects [27]. The lack of association between dissociative and antidepressant effects for uncompetitive NMDAR antagonists is supported by the rapid, robust, and sustained antidepressant effects seen with low-potency NMDAR antagonists such as the dextromethorphan-bupropion combination [17,19] and esmethadone [18], which lack dissociative effects. The early findings by Trullas and Skolnick with MK-801 involving reversal of depressive-like behavior and activation of neural plasticity [24] were subsequently also shown with ketamine [10] and the non-dissociative, low-potency, uncompetitive NMDAR antagonist esmethadone [28]. The potency of NMDAR antagonists is determined by two distinct parameters: affinity and trapping. Trapping values for ketamine and esmethadone are similar at 85.9 ± 1.9 and 86.7 ± 1.8, respectively [20]. In a similar experiment from a different study, the trapping values for ketamine and memantine were 86 ± 1 and 71 ± 4, respectively [29]. Ketamine-like affinity and ketamine-like trapping may both be required for dissociative effects [20,29]. Ketamine-like trapping, but not ketamine-like affinity, may be required for antidepressant effects [18,20]. Uncompetitive NMDAR antagonists with low affinity (e.g., esmethadone) or with low trapping (e.g., memantine) do not cause dissociative effects. Memantine, with low trapping compared to ketamine, is ineffective as an antidepressant despite ketamine-like NMDAR affinity [20,30].

## 4. NMDAR Tonic Activity and Synaptic Protein Homeostasis

Activation of neural plasticity by ketamine in models of depressive-like behavior is understood to be related to homeostatic restoration of synaptic proteins [11]. Ready availability of synaptic proteins at synapses is required for real-time stimulus-evoked neural plasticity (synaptic spine remodeling and new spine formation). NMDARs regulate Ca2+ influx in postsynaptic neurons following action potentials (phasic Ca2+ influx) and during resting membrane potentials (tonic Ca2+ influx) between phasic activations. There has recently been increased recognition of the role of tonic NMDAR-mediated postsynaptic Ca2+ currents in normal physiology and pathology. In particular, well-regulated tonic NMDAR-mediated Ca2+ influx in postsynaptic neurons during resting membrane potential was shown to be necessary to ensure the homeostatic availability of synaptic proteins [11,31,32,33,34,35]. Homeostatic availability of synaptic proteins at synapses is necessary to ensure efficient real-time stimulus-evoked neural plasticity directed by action potential-triggered, NMDAR-mediated Ca2+ influx [11,31,33,34]. If synaptic proteins are unavailable at synaptic spines, real-time efficient structural memory cannot be formed following stimulus-evoked NMDAR Ca2+ signaling at action potential [28]. In summary, the unavailability of synaptic proteins determined by upregulation of tonic Ca2+ influx through NMDARs may be the physiopathological mechanism underlying depressive-like behaviors in experimental models.

## 5. NMDAR Phasic Activity and Real-Time Stimulus-Evoked Neural Plasticity

Stimulus-evoked presynaptic glutamate release triggers phasic, action potential-mediated, postsynaptic Ca^2+^ influx through NMDARs, also defined as excitatory postsynaptic currents (eEPSCs), when glutamate reaches mM concentrations in the synaptic cleft for a few milliseconds. High glutamate concentration in the synaptic cleft activates α-amino-3-hydroxy-5-methyl-4-isoxazolepropionic acid receptors (AMPARs) in the hotspot (the postsynaptic area closest to the presynaptic site from where glutamate is released) of the postsynaptic neuron, inducing cation influx through the AMPAR channel. AMPAR-mediated membrane depolarization facilitates Mg^2+^ disengagement from the NMDAR channel pore. Mg^2+^ disengagement coincides with the glutamate-induced allosteric change of the NMDAR channel to the open configuration, determined by glutamate binding to the glutamate pocket on the NR2 subunit of the NMDAR. Therefore, during action potential, all NMDAR subtypes present at the synaptic hotspot become permeable to Ca^2+^ influx for a subtype-specific time frame that ranges from 50 milliseconds for GluN2A subtypes to a few seconds for GluN2D subtypes. In summary, action potential results in a time-regulated postsynaptic influx of Ca^2+^ via all NMDAR subtypes present at the synaptic hotspot. This phasic NMDAR activity directs real-time stimulus-evoked neural plasticity [13,14].

## 6. Graded NMDAR Tonic Activity and Stimulus-Evoked Real-Time Phasic Neural Plasticity

Efficient action potential-mediated real-time neural plasticity requires the availability of synaptic proteins at the synapse at the time of an action potential. The homeostatic availability of synaptic proteins is regulated by graded tonic Ca^2+^ influx through NMDAR channels [11,31,32,33,34,35]. Tonic Ca^2+^ influx through NMDARs at resting membrane potential is also receptor composition subtype-specific and is mediated by a small percentage of NMDAR channels in the open configuration and free of Mg^2+^ at resting membrane potential (~−80 to −50 mV). This subtype-specific tonic NMDAR Ca^2+^ permeability allows membrane potential-regulated (graded between ~−80 and −50 mV) tonic Ca^2+^ influx into the postsynaptic neuron during the periods between phasic NMDAR activation (at resting membrane potential). NMDAR Ca^2+^ permeability requires coincidental open-channel configuration and Mg^2+^ disengagement from the channel pore, not only at phasic activation but also during tonic activity. The percentage of Ca^2+^-permeable NMDAR channels at resting membrane potential increases as membrane potential depolarizes from −80 mV to −50 mV. The probability that NMDARs are Ca^2+^ permeable during tonic activity is therefore increased by “spontaneous” presynaptic release of non-depolarizing amounts of glutamate (nM concentration), also known as miniature presynaptic events (mPSEs). While the spontaneous presynaptic release of glutamate may appear stochastic, the probability of spontaneous release is likely increased by a higher density of glutamate vesicles in the presynaptic active zone. Previous subthreshold stimuli reaching the presynaptic neuron are likely to regulate the density of presynaptic glutamate vesicles ready to release glutamate upon presynaptic neuron depolarization. By definition, the amount of presynaptic glutamate released during mPSEs is non-depolarizing; otherwise, it would result in an action potential, with AMPAR-mediated depolarization, the opening of all NMDARs in the synaptic hotspot and eEPSCs. Instead, mPSEs determine miniature excitatory postsynaptic currents (mEPSCs). As membrane potential depolarizes from approximately −80 mV toward −50 mV, a progressively higher percentage of NMDARs are switched into the open configuration and disengage Mg^2+^, thus causing tonic Ca^2+^ current to increase (or to decrease in the case of membrane hyperpolarization) in a voltage- and presynaptic glutamate release (mPSEs)-dependent manner. NMDAR subtypes with a relatively higher affinity for glutamate and a relatively lower affinity for Mg^2+^ have the greatest probability of being coincidentally in the open configuration and free of Mg^2+^ during mPSEs. Thus, postsynaptic Ca^2+^ influx at resting membrane potential (−80 to −50 mV), including Ca^2+^ influx triggered by mPSEs, is mainly mediated by GluN2D channels because these subtypes have low affinity for Mg^2+^ and high sensitivity to glutamate [14,20]. These characteristics of GluN2D subtypes also result in several seconds of the open configuration at action potential instead of the 50 to 400 milliseconds open time of other subunit compositions.

In contrast with the mM concentrations of glutamate briefly reached in the synaptic cleft at action potential, the concentration of ambient glutamate at resting membrane potential resulting in measurable increases in subtype-specific NMDAR activation is as low as 40 nM [20]. The Ca^2+^ influx measured at very low ambient glutamate is likely due to preferential activation of GluN2D subtypes [20,36,37]. The same subtype, GluN2D, is also the preferential target for uncompetitive NMDAR antagonists [20,37].

## 7. Stressful Events and Glutamatergic Signaling in Health and Disease

For neurons involved in stress-related circuits, stressful events may cause an increase in ambient glutamate via mPSEs and thus increase the frequency of mEPSCs during NMDAR tonic activity. Elevated ambient glutamate increases the probability of an open configuration and Mg^2+^ disengagement from NMDARs, particularly for GluN2D receptor subtypes due to the subtype’s low affinity for Mg^2+^ and high sensitivity to glutamate. GluN2D subtypes thus preferentially contribute to tonic increases in postsynaptic Ca^2+^ influx, at or near resting membrane potential (following mEPSCs). Increased tonic Ca^2+^ influx through NMDARs halts local synaptic protein translation [11,31,34], resulting in decreased availability of synaptic proteins. The unavailability of synaptic proteins impairs stimulus-evoked neural plasticity (i.e., efficient real-time memory formation is impaired). Impairment of neural plasticity in relevant neuronal circuits affected by chronic stress contributes to the depressive-like phenotype in experimental models of depressive-like behavior and in humans subjected to stress or suffering from MDD. Uncompetitive NMDAR antagonists reverse neural plasticity impairment and depressive-like behavior in animal models [10,11,12,32,33]. Uncompetitive NMDAR antagonists reverse the depressive phenotype in patients with MDD [17,18,19,21,23]. Notably, the reversal of the depressive phenotype in patients with MDD includes improvements in subjective cognitive symptoms [38], further indicating that restoration of physiological neural plasticity may be the mechanism underlying phenotype improvement in patients, confirming results seen in experimental models [10,11,12,32,33]. In summary, finely regulated or dysregulated tonic Ca^2+^ influx through NMDARs may underlie physiological time-limited depressive behaviors and symptoms in mentally healthy individuals undergoing stressful events and may cause the more severe and persistent pathological depressive behaviors and symptoms, and cognitive deficits, in patients with MDD.

## 8. High-Potency and Low-Potency NMDAR Antagonists: The Molecular Mechanisms Underlying Dissociation and Rapid Antidepressant Effects Are Distinct

**Figure 1:**
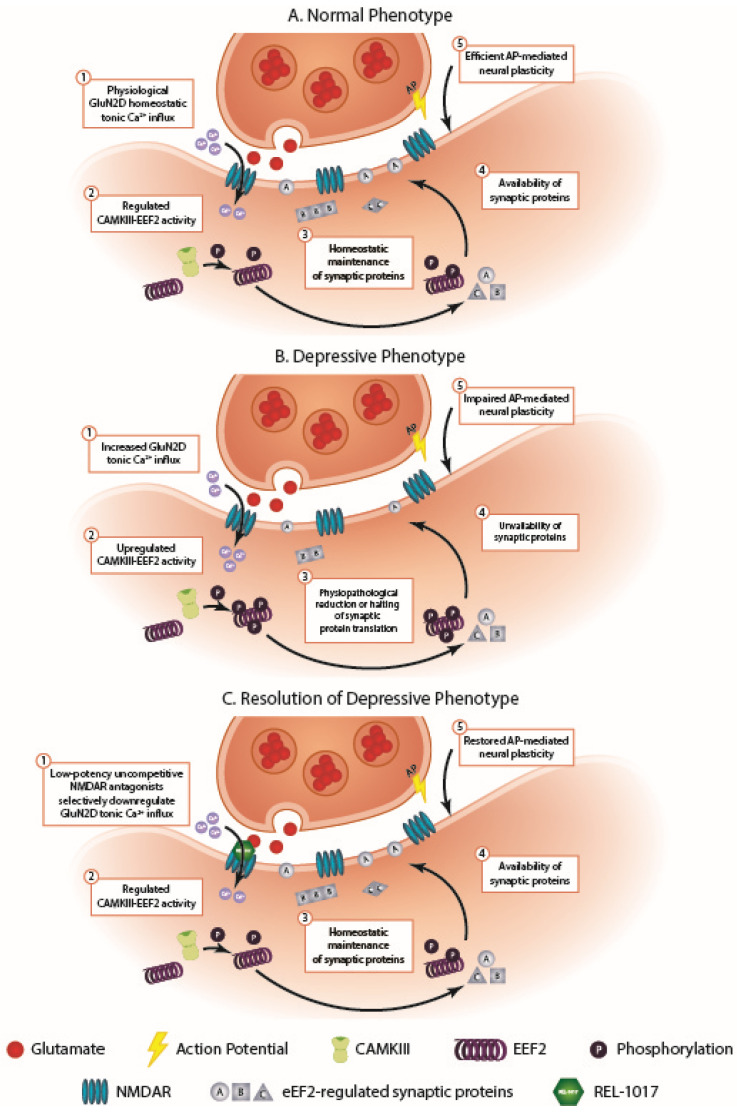
Proposed mechanism of kinase involvement in uncompetitive NMDAR antagonist-mediated rapid antidepressant effects. (**A**) In the normal phenotype, physiological GluN2D homeostatic tonic Ca^2+^ influx appropriately regulates CAMKIII phosphorylation of EEF2, which results in adequate homeostatic maintenance and availability of synaptic proteins required for action potential (AP)-mediated neural plasticity. (**B**) In the depressive phenotype, increased Ca^2+^ influx through GluN2D channels upregulates CAMKIII-EEF2 activity, leading to the halting of synaptic protein production and availability, resulting in impaired AP-mediated neural plasticity. (**C**) Resolution of the depressive phenotype is possible through the action of uncompetitive NMDAR antagonists, such as REL-1017, which block excessive tonic Ca^2+^ currents, restoring the homeostatic maintenance and availability of synaptic proteins, thereby enabling physiological AP-mediated synaptic plasticity.

Stimulus-evoked real-time synaptic remodeling (neural plasticity) is mediated by phasic (action potential-mediated) NMDAR-regulated Ca^2+^ influx into the postsynaptic neuron [13,14]. When potent NMDAR antagonists like MK-801 and phenylcyclohexyl piperidine block NMDAR channels, they block excessive tonic Ca^2+^ influx (therapeutic for MDD), but they also block phasic NMDAR transmission. The block of phasic NMDAR Ca^2+^ influx uncouples environmental stimuli from neural plasticity. Therefore, during phasic NMDAR block, environmental stimuli do not elicit efficient neuronal plasticity, causing dissociation and memory impairment. Ketamine, at standard antidepressant doses (e.g., 0.5 mg/kg IV), causes short-lived dissociation and at higher doses is a dissociative anesthetic. In contrast, the lack of dissociative effects seen in patients with MDD successfully treated with esmethadone [18] and dextromethorphan [17,19] indicates that these low-potency NMDAR antagonists do not interfere with phasic NMDAR-mediated Ca^2+^ currents at concentrations that are therapeutic for MDD, unlike racemic ketamine and esketamine at doses currently used for MDD [21,39]. Like ketamine, esmethadone blocks tonic NMDAR-mediated Ca^2+^ influx and induces brain-derived neurotrophic factor (BDNF)-dependent neural plasticity in animal models of depression, reversing depressive-like behavior [10,11,12,32]. In patients with MDD, NMDAR blockage by ketamine and esketamine temporarily interferes with phasic NMDAR activation, as clinically evidenced by dissociative symptoms in 70% of patients treated with subanesthetic, antidepressant doses of ketamine and intranasal esketamine. In contrast with the toxic temporary phasic block and neural plasticity impairment caused by ketamine (transient ketamine toxicity at C_max_), the therapeutic neural plasticity-inducing effects and sustained antidepressant effects of ketamine in murine models are similar to those induced by esmethadone [10,12] and are mediated by the block of tonic NMDAR Ca^2+^ currents, not by the block of phasic NMDAR Ca^2+^ currents [11]. As anticipated by Autry and colleagues, chronic unpredictable stress induces excessive tonic NMDAR-mediated Ca^2+^ influx, eliciting downstream events that impair the homeostatic availability of synaptic proteins, including BDNF. Figure 1 illustrates a mechanism of action hypothesis to explain the rapid antidepressant effects of esmethadone (REL-1017) and other uncompetitive NMDAR antagonists.

Physiological tonic Ca^2+^ influx through NMDARs regulates synaptic protein synthesis via the activation of eukaryotic elongation factor 2 kinase (EEF2K, or CAMKIII), which phosphorylates eukaryotic elongation factor 2 (EEF2), reducing or halting synaptic protein translation. Increased Ca^2+^ influx through NMDARs at resting membrane potential leads to enhanced activation of CAMKIII, which then drives increased phosphorylation of EEF2 and the reduction or halting of synaptic protein translation [31,34]. Uncompetitive NMDAR antagonists reduce tonic NMDAR-mediated Ca^2+^ influx and down-modulate EEF2K activity, regulating protein translation and synaptic protein availability [11]. Thus, tonic, not phasic, NMDAR block by ketamine and other uncompetitive NMDAR antagonists may result in antidepressant-like effects without compromising neural plasticity [10,12]. The activation of CAMKIII by tonic Ca^2+^ signaling through NMDARs has been established [31,34], and the role of uncompetitive NMDAR antagonists in modulating tonic Ca^2+^ currents in models of depression is increasingly understood [11,32,33,40].

The blockade of excessive tonic postsynaptic Ca^2+^ influx by uncompetitive NMDAR antagonists restores neural plasticity and determines antidepressant effects via CAMKIII kinase downregulation. The block of phasic Ca^2+^ influx by potent uncompetitive NMDAR antagonists like MK-801 results in severe dissociative side effects. The dissociative effects of ketamine and esketamine are temporarily related to C_max_ and improve and resolve within a couple of hours, while their antidepressant effects are sustained beyond receptor occupancy. This prolonged therapeutic effect supports the hypothesis that a short-lived, C_max_-dependent NMDAR phasic block causes the acute, concentration-dependent, C_max_-related, dissociative effects, while sustained antidepressant effects are determined by the blockade of tonically and pathologically open NMDARs [25] of the GluN2D subtype [20]. Dissociative effects caused by NMDAR phasic block may not be necessary for antidepressant effects of uncompetitive NMDAR antagonists, as previously suggested [22,26]. The downregulation of excessive Ca^2+^ influx restores physiological Ca^2+^ signaling and decreases the excessive activity of CAMKIII, allowing physiological protein translation, thus ensuring homeostatic synaptic protein restoration with reactivation of efficient, stimulus-evoked, phasic activity-induced neural plasticity. While CAMKIII kinase downregulation may explain the depressive phenotype, NMDAR-mediated tonic Ca^2+^ signaling may also potentially regulate other kinases in the postsynaptic density that could be important for neural plasticity.

## 9. Molecular Mechanisms Underlying Depressive Behaviors in Health and Disease

The understanding of the mechanism underlying the demonstrated clinical efficacy of uncompetitive NMDAR antagonists in patients with MDD [17,18,19,21,23,39] is rapidly progressing, supported by the understanding of the physiological and pathological roles of tonic NMDAR-mediated Ca^2+^ currents [31,34] and the results from experimental models of depressive-like behavior [10,11,12,32,33,41]. NMDARs are unique, highly regulated ion channels that, before allowing subtype-specific, time-controlled Ca^2+^ influx, require the binding of two agonist ligands, glycine and glutamate, and the coincidental disengagement of Mg^2+^ from the channel. GluN2D subtypes are the NMDAR subtypes with the highest affinity for glutamate and thus the highest probability of inhabiting the open configuration in the presence of low glutamate concentrations in the synaptic cleft [20,36]. Therefore, GluN2D subtypes are more likely to enter the open configuration when pathological ambient glutamate is present in the synaptic cleft. Additionally, GluN2D subtypes have lower affinity for Mg^2+^ as compared to other NMDAR subtypes [20,42,43,44] and thus have a higher probability of being free of Mg^2+^ when ambient glutamate is present and the receptor is in the open configuration. Another differential feature of GluN2D subtypes, compatible with their higher affinity for glutamate and lower affinity for Mg^2+^, is that they remain Ca^2+^ permeable for several seconds after an action potential, compared to 50 milliseconds for GluN2A and 300 to 400 milliseconds for GluN2C and 2B subtypes [14]. Therefore, because of their higher affinity for ambient glutamate and their lower affinity for Mg^2+^, GluN2D subtypes have a high probability of coincidental open configurations and disengagement of Mg^2+^ from the channel pore, not only after phasic activation, when all other channel subtypes are also open and free of Mg^2+^, but also during resting membrane potential. Thus, the GluN2D subtype is more likely to be tonically permeable to Ca^2+^ influx in the presence of ambient glutamate. The tonic Ca^2+^ permeability of this synaptic receptor subtype may therefore be pivotal in understanding “physiological” depressive behavior (after a stressful event) and pathological depressive behavior (during a major depressive episode).

Tonic Ca^2+^ currents via GluN2D subtypes are well described, and their physiological role in neural plasticity is actively studied [45]. As Autry and colleagues showed in depressive-like animal behavioral models, excessive tonic NMDAR-mediated Ca^2+^ influx impairs the availability of synaptic proteins at the synapse [11]. The reduction of mEPSC by ketamine (i.e., the reduction of tonic Ca^2+^ currents via NMDARs elicited by “spontaneous” mPSEs) potentiates subsequent evoked synaptic responses, i.e., phasic activity mediated by AMPAR activation and depolarization [11,32]. These experimental results support a therapeutic mechanism of action for uncompetitive NMDAR antagonists consisting of enhanced action potential-mediated synaptic plasticity dependent on the downregulation of excessive tonic Ca^2+^ currents that direct synaptic protein availability, including BDNF. This mechanism has been shown in similar experimental models for ketamine [10,11,32,33] and esmethadone [12]. Notably, the BDNF gene has been implicated in the therapeutic effects of ketamine in experimental models of depressive-like behavior [10,11,12] and patients with MDD [46]. Esmethadone and ketamine also increased BDNF levels in healthy subjects [47,48]. Ketamine increased BDNF in depressed patients [49] and may not be effective in MDD patients with BDNF gene mutations [50]. Recent experimental work suggests that interactions between the NMDAR system and the endorphin system are necessary to achieve antidepressant effects from ketamine; however, ketamine’s antidepressant effects are not mediated by opioid agonist effects [41]. Because of similar postulated mechanisms of action of ketamine and esmethadone in experimental models of depressive-like behavior [10,12], it is conceivable that a functional endorphin system may also be necessary for the therapeutic effects of esmethadone and other uncompetitive NMDAR antagonists.

## 10. Hyperactivation of GluN2D Subtypes and Major Depressive Disorder

Physiological tonic Ca^2+^ influx through GluN2D subtypes may become excessive under pathological circumstances, such as in the presence of chronic low-concentration ambient glutamate in the synaptic cleft, pathological agonists, or positive allosteric modulators (PAMs). Therefore, GluN2D subtypes may be a preferential pathological target in situations of excessive presynaptic glutamate release, in situations of impaired glutamate clearance [51], or in the presence of pathological agonists or pathological PAMs [52]. The same GluN2D receptor is also the preferential therapeutic target for uncompetitive NMDAR antagonists in the presence of physiological Mg^2+^ [20,37]. Furthermore, like the NMDAR channel block exerted by Mg^2+^, the channel block exerted by esmethadone depends on membrane potential [20]. At physiological pH, esmethadone is positively charged [53]; therefore, like Mg^2+^, esmethadone is retained in the NMDAR channel when the membrane potential is negative [20]. In contrast, during stimulus-evoked phasic NMDAR activation and AMPAR-mediated membrane depolarization, both Mg^2+^ and esmethadone are disengaged from the channel, allowing physiological decoding of incoming stimuli via Ca^2+^ quanta that are tightly regulated by NMDARs. The lack of esmethadone blocks of phasic NMDAR activation explains the absence of dissociative effects at concentrations therapeutic for MDD. Dissociative effects associated with the phasic block of NMDARs are not necessary for the antidepressant effects of ketamine. The antidepressant effects only require a tonic block of excessively open GluN2D subtypes. Ketamine is also positively charged [54]; nevertheless, it causes temporary dissociative effects in a high percentage of patients at C_max_ reached by antidepressant doses in current use, similarly to esketamine. The 10-fold higher affinity at the NMDAR of ketamine and esketamine, compared to esmethadone [20], narrows their safety window because of phasic block of NMDARs at C_max_ reached by antidepressant doses in current use. Arketamine, because of its four-fold lower potency compared to esketamine, may have the potential to relieve depressive symptoms without causing dissociative side effects [55], similarly to the lower-potency NMDAR l antagonists esmethadone and dextromethorphan [17,18]. Interestingly, the sustained antidepressant-like effects of arketamine are lost in GluN2D subunit knockout (GluN2D-KO) mice [56]. This experimental finding supports that arketamine may exert sustained antidepressant-like effects by preferential block of GluN2D subtypes, as postulated for other low-potency NMDAR antagonists, including esmethadone.

Low-potency uncompetitive NMDAR antagonists may relieve depression by preferentially blocking open GluN2D channels. In this hypothesis, depression may be primarily caused by tonically and pathologically open GluN2D channels with chronic excessive Ca^2+^ influx through this NMDAR subtype. GluN2D channel subtypes are the preferential target for low glutamate concentrations in the synaptic cleft [20,51] and are the therapeutic target for uncompetitive NMDAR antagonists [20,37]. The physiological role of tonic Ca^2+^ currents in the regulation of synaptic protein availability has been anticipated by Sutton [31,34]. Hanson and colleagues have highlighted the importance of the GluN2D receptor subtype in neuronal plasticity [45], and experimental work has implicated GluN2D in emotional memory processing [51]. Experimental work by Autry and colleagues indicates that excessive tonic NMDAR-mediated Ca^2+^ influx is associated with excessive EEF2K (CAMKIII) activation and suppression of synaptic protein availability. The blockade of excessive tonic Ca^2+^ currents lead to subsequent EEF2K downregulation. Downregulation of EEF2K results in de-suppression of protein translation and homeostatic synaptic protein restoration, including BDNF, and restoration of phasic, action potential-mediated neural plasticity, leading to antidepressant-like effects [11].

## 11. Conclusions

The sustained antidepressant effects of esmethadone [18] and other low-potency NMDAR antagonists that are effective against MDD at non-dissociative doses, such as dextromethorphan [17,19], and potentially arketamine [55], may be determined by a preferential block of tonically and pathologically hyperactive GluN2D subtypes at resting membrane potential. This tonic block does not extend to other receptor subtypes, which have a higher probability of closed configuration and Mg^2+^ engagement at resting membrane potential. Furthermore, this tonic block by low-potency uncompetitive NMDAR antagonists does not extend to phasic NMDAR activity. This preferential tonic block of GluN2D subtypes is shared by all uncompetitive NMDAR antagonists, although the more potent antagonists, such as racemic ketamine and esketamine, lack selectivity for tonically and pathologically hyperactive GluN2D subtypes at doses in clinical use for MDD. The interference with phasic NMDAR activity determined by potent NMDAR antagonists manifests as dissociative effects (at C_max_). Temporary dissociative effects peak around the C_max_ reached by doses of ketamine and esketamine currently used in MDD treatment. Dissociative effects can therefore be considered C_max_-related side effects of the more potent NMDAR antagonists, due to their narrower safety window compared to lower-potency uncompetitive NMDAR antagonists. Dissociative side effects are not necessary for the NMDAR antagonist therapeutic effects in MDD and may be detrimental for patients, not only acutely but also chronically. Higher-potency NMDAR antagonists cause Olney’s lesions in rats [57,58,59,60]. While these neuropathological rat findings are of unclear significance for human safety, they cannot be discounted. Chronic ketamine abuse has been associated with brain damage and cognitive impairment [61], and similar effects cannot be excluded when ketamine is used chronically at dissociative doses for MDD treatment. In contrast, esmethadone and racemic methadone do not cause Olney’s lesions in rats [62]. Chronic use of racemic methadone, a mu-opioid agonist and low-potency NMDAR antagonist [63], is not directly associated with brain changes or cognitive impairment and has been associated with improved cognition [64]. Therefore, the chronic use of esmethadone and other low-potency uncompetitive NMDAR antagonists may be safer compared to the chronic use of more potent NMDAR antagonists, such as ketamine and esketamine. Similar to ketamine, esmethadone increases levels of synaptic proteins and induces rapid antidepressant actions through BDNF-mediated synaptic plasticity in animal models [12]. In summary, the mechanism of disease for MDD may be tonic hyperactivity of GluN2D subtypes in MDD-relevant neuronal circuits, and the mechanism of action for uncompetitive NMDAR antagonists for the treatment of MDD may be tonic block of hyperactive GluN2D subtypes. These paradigm-changing hypotheses, if supported by future experiments, may advance our understanding of the pivotal role of NMDARs in health and disease. Additional experimental work directly linking esmethadone to modulation of EEF2K via GluN2D antagonism is needed.

## Data Availability

Not applicable.
